# Association between APOC3 polymorphisms and non-alcoholic fatty liver disease risk: a meta-analysis

**DOI:** 10.4314/ahs.v20i4.34

**Published:** 2020-12

**Authors:** Jun Wang, Chuncui Ye, Sujuan Fei

**Affiliations:** Department of Gastroenterology, the Affiliated Hospital of Xuzhou Medical University, Xuzhou 221002, Jiangsu Province, China

**Keywords:** Apolipoprotein C3, polymorphism, non-alcoholic fatty liver disease, meta-analysis

## Abstract

**Background and Aim:**

The apolipoprotein C3 (APOC3) polymorphism has been reported to predispose to non-alcoholic fatty liver disease (NAFLD). However, the results remain inconclusive. This meta-analysis aimed to provide insights into the association between APOC3 polymorphisms and NAFLD risk.

**Methods:**

Studies with terms “NALFD” and “APOC3” were retrieved from PubMed, Web of Science, CNKI and Wanfang databases up to August 1, 2019. Pooled odds ratio (OR) and 95% confidence interval (95% CI) for the association of APOC3 polymorphisms and NAFLD risk were calculated using fixed and random-effects models.

**Results:**

A total of twelve studies from eleven articles were included. Of them, eight studies (1750 cases and 2181 controls) reported the strong association of variant rs2854116 with NAFLD and six studies (1523 cases and 1568 controls) found the association of rs2854117 polymorphism with NAFLD. Overall, a statistically significant association between rs2854116 polymorphism of APOC3 gene and NAFLD risk was found only under dominant model. However, association of rs2854117 polymorphism with NAFLD risk was not detected under all four genetic models. In sub-group analysis of NAFLD subjects based on country, no association among them in China was detected. Besides, four studies analyze the association between the two polymorphisms and clinical characteristics in all subjects or NAFLD patients, and we also failed detect any association between the wild carriers and variant carriers.

**Conclusion:**

The meta-analyses suggests that the rs2854116 polymorphism but not rs2854117 polymorphism in APOC3 gene might be a risk factor for NAFLD among Asians. That is, individuals with CT+CC genotype have higher risk of developing NAFLD. However, studies with sufficient sample size are needed for the further validation.

## Introduction

Non-alcoholic fatty liver disease (NAFLD) is one of the most common cause of liver disease worldwide with prevalence varying from 15% to 40% in general population depending on the demographic area 1. Currently, the population prevalence in Asia is around 25%. Among more affluent regions of China, the community prevalence is about 15% 2,3. The spectrum of this disease ranges from bland steatosis, non-alcoholic steatohepatitis (NASH), fibrosis, cirrhosis, and occasionally to hepatocellular carcinoma 4 . NAFLD is considered to be the hepatic manifestation of the metabolic syndrome (MS), which is characterized by obesity, type 2 diabetes, dyslipidemia and hypertension with insulin resistance being the main mechanisms 5. Although the exact etiology of NAFLD is not well delineated, it is a complex metabolic condition in which both lifestyle and genetic factors play a pathogenic role 6.

The apolipoprotein C3 gene (APOC3) is a member of the APOA1/C3/A4/A5 gene cluster and located on chromosome 11q23, an area in strong linkage with lipid metabolism 7. APOC3 is a 79-amino-acid glycoprotein and a major component of TG-rich lipoproteins (TRLs). Indeed, APOC3 impairs the lipolysis of TRLs by inhibiting lipoprotein lipase and the hepatic uptake of TRLs by remnant receptors 8,9. A few genetic studies suggest that the single nucleotide polymorphisms (SNPs) in the APOC3 gene may have implications for hypertriglyceridemia 10. Homozygotes for the C-482T and T-455C variants are resistant to insulin-mediated down-regulation of APOC3 gene transcription, which results in high TG levels 11. Recently, several studies have investigated the association between single nucleotide polymorphisms (SNPs) in the APOC3 gene and NAFLD risk. Of those SNPs, two were most commonly investigated: rs2854116 (T455C in the promoter region), and rs2854117 (C482T in the promoter region). However, the results have been inconsistent 12–25. In this study, we conducted a meta-analysis in order to get a robust conclusion about the association between the two polymorphisms in the APOC3 gene and NAFLD susceptibility.

## Materials and Methods

### Search strategy

We searched the PubMed, Web of Science, CNKI and Wanfang database before August 1, 2019, by using the key subjects “apolipoprotein C3 or APOC3” AND “genetic polymorphism or polymorphisms or variant” AND “non-alcoholic fatty liver disease or NAFLD”. Studies were restricted to human populations and studies written in other languages except for English were also considered. Additional studies were identified by a hand search of references of original or review articles on this topic.

### Inclusion criteria and exclusion criteria

Studies were included according to the following criteria: (1) studies with clear diagnostic criteria, (2) studies that evaluatd the association between the APOC3 polymorphisms and NAFLD, (3) studies in a case-control design, and (4) studies with detailed genotype frequency of cases and controls or providing the necessary data to calculate genotype frequency. Studies were excluded when they were: (1) case-only study, case reports, and review articles, (2) studies without the raw data of the APOC3 genotype, and (3) repetitive publications.

### Data extraction

For each study, the following data were extracted independently by two investigators: the first author's name, year of publication, country of origin, age, gender, study design, diagnostic criteria of NAFLD, number of cases and controls, and HWE in controls (P value) and analysis were carried on independently. The results obtained by those two investigators were compared, and disagreements were discussed among all authors and resolved with consensus.

## Statistical analysis

The risk of NAFLD associated with the two polymorphisms of the APOC3 gene was estimated for each study by odds ratio (OR) and 95% confidence interval (95%CI). A χ2-test-based Q statistic test was performed to assess the between-study heterogeneity 26. We also quantified the effect of heterogeneity by I2 test. When a significant Q test (P≤ 0.05) or I2 >50% indicated heterogeneity across studies, the random effects model was used 27. Otherwise, the fixed effects model was adopted 28. Before estimating the effect of APOC3 polymorphisms on NAFLD, we tested whether genotype frequencies of controls were in HWE using χ2 test. We first estimated the risks of the heterozygote and variant homozygote compared with the wild-type homozygote, respectively, and then evaluated the risks of the combined variant homozygote and heterozygote versus the wild-type homozygote, and the variant homozygote versus the combined heterozygote and wildtype homozygote, assuming dominant and recessive effects of the variant allele, respectively. We performed stratification analyses on country. Analysis of sensitivity was performed to evaluate the stability of the results. Finally, potential publication bias was investigated using Begg' funnel plot and Egger's regression test 29,30. P<0.05 was regarded as statistically significant.

All statistical analyses were performed using software Cochrane Collaboration RevMan 5.2 and STATA package version 12.0 (Stata Corporation, College Station, Texas).

## Results

### Study characteristics

**Figure 1:**
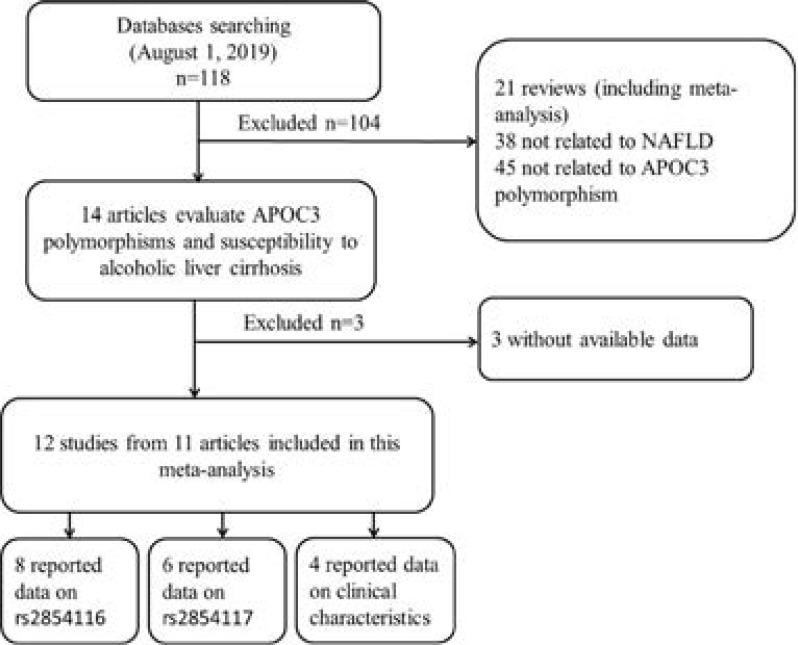
Flow chart showing study selection procedure.

After an initial search, a total of 118 published articles relevant to the topic were identified. According to the inclusion criteria, 15 studies with full-text were included in this meta-analysis and 103 studies were excluded. Because the study by Cai et al 12 included two populations, we treated them separately in this meta-analysis. We excluded three studies 23–25 due to the absence of detailed genotyping information. The process of study selection is briefly summarized in Figure 1. As shown in Table 1, there were 8 case-control studies with 1750 NAFLD cases and 2181 controls concerning rs2854116 polymorphism and 6 case-control studies with 1523 cases and 1568 controls concerning rs2854117 polymorphism. Four studies with 2384 subjects reported the clinical and biochemical parameters in the study grops. The distribution of genotypes in the controls was consistent with the Hardy-Weinberg equilibrium for almost all selected studies except one study^19^.

**Table 1:** Characteristics of studies included in the meta-analysis

Study	Year	Country	Age, mean ± SD, year		Gender(male/female)		Diagnostic criteria	Genotype (case/control)			P_HWE_
		
			case	control	case	control		WT Ho	Ht	VR Ho	
**rs2854116**								TT	TC	CC	
Cai *et al.*	2013	China (Han)	43.68±10.72	42.88±10.0	NA	NA	The criteria of NAFLD designed by the Chinese Liver Disease Association (2010)	51/70	109/103	33/38	0.992
Cai *et al.*	2013	China (Uyghur)	42.39±8.79	41.59±9.07	NA	NA	The criteria of NAFLD designed by the Chinese Liver Disease Association (2010)	56/54	115/94	32/40	0.939
Li M *et al.*	2014	China	40.7±9.7	39.5±9.1	200/100	200/100	Ultrasonography	94/134	131/123	75/43	0.093
Niu *et al.*	2014	China	49.76±16.17	47.69±15.86	175/215	198/211	The criteria of NAFLD designed by the AASID	102/104	180/195	108/110	0.350
Niu *et al.*	2014	China	49.96±16.20	47.81±16.19	132/155	146/164	The criteria of NAFLD designed by the Chinese Liver Disease Association (2010)	81/80	139/147	67/83	0.364
Puppala *et* *al.*	2014	India	44.1±12.1	42.6±10.6	92/58	92/58	Ultrasonography	44/60	75/81	31/9	0.007
Song *et al.*	2017	China	NA	NA	39/93	94/158	The criteria of NAFLD designed by the Chinese Liver Disease Association (2010)	44/88	63/117	23/46	0.517
Yang *et* *al.*	2018	China	70.95±4.73	72.53±5.67	25/72	133/229	Guidelines for management of nonalcoholic fatty liver disease: an updated and revised edition	39/135	46/177	12/50	0.506
**rs2854117**											
Cai *et al.*	2013	China (Han)	43.68±10.72	42.88±10.0	NA	NA	The criteria of NAFLD designed by the Chinese Liver Disease Association (2010)	52/71	106/99	35/41	0.539
Cai *et al.*	2013	China (Uyghur)	42.39±8.79	41.59±9.07	NA	NA	The criteria of NAFLD designed by the Chinese Liver Disease Association (2010)	73/71	105/86	25/31	0.567
Li *et al.*	2014	China	40.7±9.7	39.5±9.1	200/100	200/100	The criteria of NAFLD designed by the Chinese Liver Disease Association (2010)	108/126	144/127	48/47	0.118
Niu *et al.*	2014	China	49.76±16.17	47.69±15.86	175/215	198/211	The criteria of NAFLD designed by the AASID	107/104	176/203	107/102	0.882
Niu *et al.*	2014	China	49.96±16.20	47.81±16.19	132/155	146/164	The criteria of NAFLD designed by the Chinese Liver Disease Association (2010)	81/79	132/156	74/75	0.907
Puppala *et* *al.*	2014	India	44.1±12.1	42.6±10.6	92/58	92/58	Ultrasonography	55/62	57/46	38/42	0.000

HWE, Hardy-Weinberg equilibrium; *P*_HWE_ was calculated by goodness-of fit *χ*^2^-test, *P*_HWE_ < 0.05 was considered statistically significant; NA, not available. Ht, heterozygote; VR Ho, variant homozygote; WT Ho, wide-type homozygote.

### Quantitative synthesis

**Figure 2:**
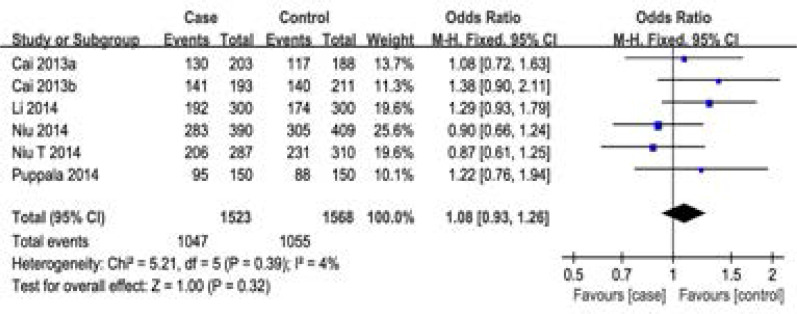
Meta-analysis of the association between rs2854116 polymorphism and susceptibility to NAFLD under dominant model.

**Figure 3:**
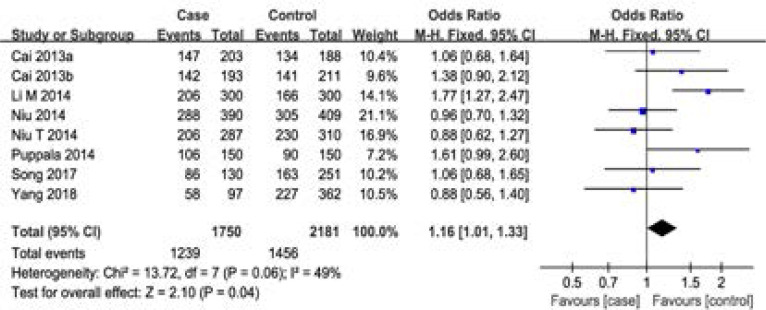
Meta-analysis of the association between rs2854117 polymorphism and susceptibility to NAFLD under dominant model.

For rs2854116 polymorphism, 8 case-control studies with 1750 cases and 2181 controls were identified. Overall, a significant association was detected under dominant model (OR=1.16, 95%CI: 1.01–1.33, P=0.04), however, there was no significant difference in APOC3 genotype distribution between NAFLD and control (OR=1.14, 95%CI: 0.82–1.57, P=0.44 for recessive model, OR=1.13, 95%CI: 0.98–1.31, P=0.10 for CT vs TT, OR=1.24, 95%CI: 0.84–1.82, P=0.28 for CC vs TT) (Figure 2, Table 2). That is to say, individuals with TC or CC genotype had a higher risk of developing NAFLD. In the sub-group analysis by country, no significant association was found in Chinese populations. Although only one study focused on Indian population, our result suggested that the APOC3 gene polymorphism T-455C (rs2854116) was significantly associated with NAFLD. For rs2854117 polymorphism, 6 case-control studies with 1523 cases and 1568 controls were identified. Overall, no significant association between the polymorphism of APOC3 gene and NAFLD risk was detected (OR=1.08, 95%CI: 0.93–1.26 for dominant model, OR=1.00, 95%CI: 0.84–1.19 for recessive model, OR=1.10, 95%CI: 0.93–1.29 for CT vs CC, OR=1.02, 95%CI: 0.84–1.25 for TT vs CC) (Figure 3, Table 2). No correlation was detected between groups stratified on country among Chinese populations. Furthermore, one study indicated that the C-482T polymorphism was not a risk indicator of NAFLD for Indian.

**Table 2:** Summary of ORs of the APOC3 polymorphisms and NAFLD risk

SNP	N	Dominant model			Recessive model			Ht *vs.* WT Ho			VR Ho *vs.* WT Ho		
					
		OR(95% CI)	P^a^	I^2^	OR(95% CI)	P^a^	I^2^	OR(95% CI)	P^a^	I^2^	OR(95% CI)	P^a^	I^2^
rs2854116	8	1.16(1.01,1.33)	0.06	49%	1.14(0.82,1.57)	0.0007	72%	1.13(0.98,1.31)	0.44	0%	1.24(0.84,1.82)	0.0003	74%
rs2854117	6	1.08(0.93,1.26)	0.39	4%	1.00(0.84,1.19)	0.76	0%	1.10(0.93,1.29)	0.16	38%	1.02(0.84,1.25)	0.93	0%

N: number of studies

a Test for heterogeneity.

^#^ Random-effect model was used when the P for heterogeneity test was ≤. 0.05, otherwise the fixed-effect model was used. CI, confidence interval; OR, odds ratio; SNP, single-nucleotide polymorphism; Ht+VR Ho *vs.* WT Ho, dominant model; VR Ho *vs.* Ht+WT Ho, recessive model.

**Figure 4:**
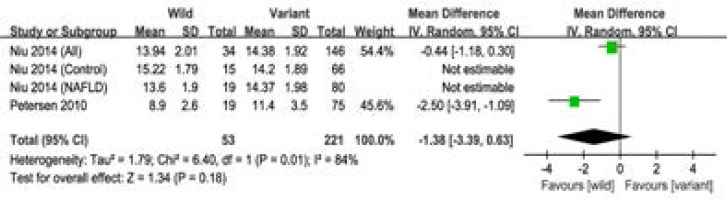
Meta-analysis of the association between the two polymorphisms and the APOC3 level.

In addition, four studies with 2384 subjects reported the clinical characteristics in the study groups. No significant association was observed between the polymorphism and clinical characteristics (BMI, Waist, APOC3, AST, ALT, FINS, HOMA-IR, FPG, TG, HDL, LDL, TC) (Figure 4, Table 3). Moreover, three studies with 1148 subjects reported the clinical characteristics in the NAFLD group, and Our results suggested the polymorphism was not associated with the clinical characteristics (BMI, HOMA-IR, FPG, TG, HDL, LDL) (Table 3).

**Table 3:** Summary of SMD of the clinical characteristics between the wild carriers and variant carriers

	All subjects				NAFLD group			
		
	N	SMD(95% CI)	P^a^	I^2^	N	SMD(95% CI)	P^a^	I^2^
BMI	5	0.16(-0.25,0.58)	0.86	0	3	0.48(-0.04,0.99)	0.83	0
Waist	2	-0.09(-3.48,3.30)	0.16	50				
FPG	5	-1.18(-2.94,0.58)	0.42	0	3	-0.49(-4,27,3.29)	0.19	40
HOMA-IR	4	-0.06(-0.13,0.00)	0.29	19	3	-0.11(-0.22,0.01)	0.60	0
TG	6	-4.50(-19.36,10.35)	0.005	70	3	2.69(-0.97,15.10)	0.20	38
HDL	6	0.83(-0.68,2.34)	0.19	33	3	-3.09(-8.17,1.99)	0.003	83
LDL	6	1.81(-6.09,9.71)	0.004	72	3	4.02(-2.39,10.43)	0.57	0
TC	3	1.37(-11.98,14.73)	0.01	77				
FINS	2	-0.06(-0.26,0.13)	0.42	0				
APOC3	2	-1.38(-3.39,0.63)	0.01	84				
ALT	2	-0.01(-4.88,4.85)	0.99	0				
AST	2	-2.13(-5.65,1.38)	0.47	0				

N: number of studies

a Test for heterogeneity

BMI, body mass index; FPG, fasting blood glucose; HOMA-IR, homeostatic model assessment of insulin resistance; TG, triglyceride; HDL, high-density lipoprotein; LDL, low-density lipoprotein; TC, total cholesterol; FINS, fasting blood insulin; APOC3, apolipoprotein C3; ALT, alanine aminotransferase; AST, aspartate aminotransferase; NAFLD, nonalcoholic fatty liver disease; SMD, standardized mean difference

### Heterogeneity and sensitivity analysis

For rs2854116 polymorphism, significant heterogeneity between studies was observed in overall comparisons under recessive and CC versus TT models (I2=72%, Pheterogeneity=0.0007, I2=74%, Pheterogeneity= 0.0003, respectively). For rs2854117 polymorphism, no significant heterogeneity between studies was observed in overall comparisons. Then, sensitivity analysis, after removing one study at a time, was performed to evaluate the stability of the results. The results was not changed when any single study was omitted, confirming the stability of the results.

### Publication bias

**Figure 5:**
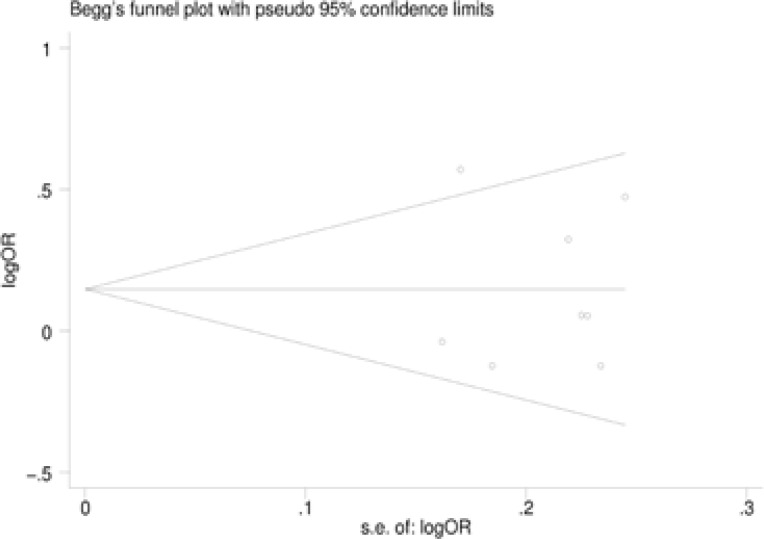
Begg's funnel plot for publication bias under dominant model. **a** rs2854116. **b** rs2854117.

Begg's funnel plot and Egger's test were performed to assess the potential publication bias in the available literature. The shape of funnel plots did not reveal any evidence of funnel plot asymmetry (Figure 5). Egger's test also showed that there was no statistical significance for the evaluation of publication bias under dominant model (rs2854116: P=0.987, rs2854117: P=0.383).

## Discussion

NAFLD is one of the most common liver disorders^31^,^32^. Although the pathogenesis of NAFLD remains largely unknown, insulin resistance, oxidative stress and inflammation play important roles in the development and progression of NAFLD 33. Many candidate genes were reported to be associated with NAFLD risk, such as TNF-α, PNPLA3, APOC3, PPAR-γ and adiponectin^34^–^37^. Our previous study^38^ performed a meta-analysis to evaluate the association between adiponectin polymorphisms and NAFLD susceptibility and the result suggested that adiponectin +45T>G and −11377C>G polymorphisms might be a risk factor for NAFLD, and +276G>T polymorphism may be a protective factor for NAFLD among Asians. As for APOC3, it is a glycoprotein synthesized mainly in the liver and the intestinal, and plays an essential role in regulating the serum triglyceride levels. Two polymorphisms, T-455C and C-482T, were located in the 5'promoter region and were in a strong linkage disequilibrium with each other. This polymorphism leads to a roughly 30% higher plasma concentration of ApoC3, and postprandial hypertriglyceridaemia. As a result, individuals with carrier of those polymorphisms can take up increased amounts of lipid from the chylomicron remnant, leading to NAFLD and hepatic insulin resistance 39. Transgenic mice overexpressing human APOC3 were predisposed to hepatic steatosis, indicating that APOC3 might play an important role in the development of NAFLD 40,41. However, the results remain controversial. Although Petersen et al. have recently reported that the polymorphisms C-482T and T-455C in APOC3 are associatd with nonalcoholic fatty liver disease and insulin resistance, the association of APOC3 variants and NAFLD risk has not yet been validated by others so far.

Recently, the APOC3 polymorphisms have been investigated the association with many diseases, such as metabolic syndrome 42, type 2 diabetes 43, coronary heart disease 44 and plasma APOC3 and lipid levels 45. As for NAFLD, a previous meta-analysis conducted by Zhang et al. 46, evaluated the association between APOC3 polymorphisms and risk of NAFLD based on 7 studies and reported that the APOC3 gene polymorphism is not a genetic risk factor for NAFLD. Ithis study, we conducted a comprehensive literature search in different databases and included several additional studies, which allowed for a larger number of subjects (12 studies) and more precise risk estimation. Besides, we further analyze the association between the two polymorphisms and clinical characteristics in all subjects or NAFLD patients. A significant association between the rs2854116 polymorphism of APOC3 gene and NAFLD risk were found under dominant model. However, no association of rs2854117 polymorphism with NAFLS was detected. When stratified by country, no association among them in China was detected. Interestingly, Puppala et al suggested that the APOC3 gene polymorphism T-455C (rs2854116) was significantly associated with NAFLD in Southern Indian population. These inconsistent results may be attributed to differences in genetic backgrounds, environmental factors, and other factors, such as small sample size or inadequate adjustment for confounding factors.

In this(our) study, the potential correlation was also analyzed between the two polymorphisms and clinical characteristics. Four studies were enrolled and the combined results showed that there was no significant difference between the wide-type genotype and the variant genotypes in either all study groups or NAFLD group. Since only few studies reported the available data, this result should be interpreted with caution, and more studies are needed.

Heterogeneity between studies is very common in the meta-analysis of genetic association studies 47. In this(our) study, heterogeneity was found under recessive model and CC versus TT model for rs2854116 polymorphism. So stratified analysis by country was performed and the heterogeneity also existed in Asian population. Then sensitivity analyses were conducted by successively excluding one study, and the estimated pooled OR changed quite little, strengthening the conclusions from this meta-analysis. Besides, no publication bias was not observed, backuping the validity and generalization of our conclusions.

Some limitations of this meta-analysis should be addressed. First, due to incomplete raw data or publication limitations, some relevant studies could not be included in our analysis. Second, the number of published studies, especially for Indian population was not sufficiently large for a comprehensive analysis, and some studies with small sample size may not have enough statistical power to explore the real association. Third, our results were based on unadjusted estimates, and lacking of the necessary information (such as age, gender, family history and other risk factors) for the date analysis may cause serious confounding bias.

In summary, this meta-analysis suggested that APOC3 rs2854116 polymorphism is associated with NAFLD risk. Individuals with CT+CC genotype had an increased risk of NAFLD. Studies with large sample zise are needed to validate our findings.
